# Access to and Use of Hand Hygiene Resources during the COVID-19 Pandemic in Two Districts in Uganda, January–April 2021

**DOI:** 10.4269/ajtmh.23-0031

**Published:** 2023-08-28

**Authors:** Caroline Pratt, Maureen Kesande, Fred Tusabe, Alexandra Medley, Graeme Prentice-Mott, Matthew Lozier, Victoria Trinies, Sauda Yapswale, Stella Nabatyanga, Herbert Isabirye, Mohammed Lamorde, David Berendes

**Affiliations:** ^1^ Waterborne Disease Prevention Branch, Division of Foodborne, Waterborne, and Environmental Diseases, U.S. Centers for Disease Control and Prevention, Atlanta, Georgia;; ^2^ U.S. Epidemic Intelligence Service, U.S. Centers for Disease Control and Prevention, Atlanta, Georgia;; ^3^ Infectious Disease Institute, Makerere University, Kampala, Uganda;; ^4^ Division of Foodborne, Waterborne, and Environmental Diseases, U.S. Centers for Disease Control and Prevention, Atlanta, Georgia

## Abstract

To understand access to and use of hand hygiene in healthcare facilities (HCFs) and community locations during the COVID-19 pandemic, we evaluated factors associated with hand hygiene in 60 priority HCFs and community locations in two border districts in Uganda. We assessed water and hand hygiene resource availability and observed hand hygiene practice by staff or patrons. Regression modeling estimated factors associated with the availability or use of hand hygiene. In HCFs, most inpatient (61%), outpatient (71%), and laboratory or staff (90%) rooms contained hand hygiene materials. Only 38% of community locations had hand hygiene materials at all entrances and exits, 35% of congregation areas had hand hygiene materials. Overall, 38% of healthcare staff, 48% of patrons post-latrine use, and 21% of patrons entering or exiting community locations practiced hand hygiene. HCF hand hygiene access was lower in inpatient rooms (odds ratio [OR] = 0.17, 95% CI: 0.06–0.45) and outpatient rooms (OR = 0.23, 95% CI: 0.07–0.70) compared with laboratory/staff rooms. HCF hand hygiene practice was higher for doctors than nurses (OR = 3.58, 95% CI: 1.15–11.14) and with new versus existing patient encounters (OR = 2.27, 95% CI: 1.20–4.27); it was lower before versus after patient contact for both invasive (OR = 0.03, 95% CI: 0.00–0.20) and noninvasive (OR = 0.66, 95% CI: 0.45–0.95) procedures. In community settings, hand hygiene practice after using the latrine was higher than at an entrances/exits (OR = 3.39, 95% CI: 2.08–5.52). Hand hygiene rates were relatively low in healthcare and community settings. Greater emphasis on hand hygiene before patient interactions (at HCFs) and at community entrances/exits for patrons is also needed.

## INTRODUCTION

Consistent and properly performed hand hygiene is a critical nonpharmaceutical intervention for preventing communicable diseases globally and is integral to mitigating the global COVID-19 pandemic, especially in low- and middle-income countries (LMICs) with low vaccine rates.^1^^,^^2^ Existing assessments show low availability of hand hygiene resources in rural Ugandan healthcare facilities (HCFs) and schools.^3^ Essential to quality healthcare, basic water services (water from an improved source available on-site) were present in 51% of HCFs in sub-Saharan Africa in 2019 and 61% in Uganda in 2022.^4^^,^^5^ In 2019, 30% of schools in Uganda had basic hygiene services (at least one handwashing station with water and soap), 24% had limited hygiene services (handwashing station with water but no soap), and 46% had no hygiene services (no handwashing station or no water).^3^ Assessments of hand hygiene in other community locations, such as community congregation points of elevated importance (e.g., markets, points of entry) during the pandemic^6^ were rare.

In addition to handwashing stations, alcohol-based hand rub (ABHR) can also be used for hand hygiene in many settings, but availability is limited in LMICs due to high commercial prices during the pandemic and limited local production.^7^^,^^8^ As demonstrated during Ebola virus disease outbreaks, local production using WHO formulas^9^^,^^10^ can help provide affordable and readily available ABHR.^11^

Although robust guidelines exist for hand hygiene access and use in HCFs,^9^^,^^12^ fewer guidelines exist for community (non-healthcare, public) settings, where hand hygiene is still critical to preventing the spread of respiratory and enteric diseases in spaces where people gather.^13^ Even when hand hygiene materials are available in HCFs, their use is often inconsistent: a 2014 survey of HCFs in Bangladesh found 69% of hospitals had hand hygiene at points of care, but only 17% of healthcare workers (HCWs) washed hands after patient contact and just 2% before patient contact.^4^ In a school-based intervention assessment in rural Uganda, students reporting “always” washing their hands at school increased markedly after receiving handwashing stations,^14^ soap, and a handwashing education program.^15^ In addition to capability and opportunity, motivation is also a key factor for why individuals do or do not perform behaviors like hand hygiene.^16^

To gain a better understanding of access to hand hygiene infrastructure and adherence to recommended hand hygiene in healthcare and community settings in the pandemic context, as well as inform mitigation and response measures, we evaluated priority HCF and community locations with potentially high risk for COVID-19 transmission in two border districts in Uganda in January–April 2021. Results will provide insight into access to and use of hand hygiene in LMICs during the COVID-19 pandemic and identify gaps in access and use in HCFs and community settings with a high risk of population mixing, which take on enhanced importance during respiratory outbreaks and the current pandemic.

## MATERIALS AND METHODS

### Locations.

Amuru (in northwestern Uganda) and Tororo (in eastern Uganda) are border districts that were targeted for COVID-19-focused mitigation activities due to high volumes of trucking and mobile populations that yielded concern for disease transmission. In these districts, 60 locations were identified as a priority for hand hygiene assessment due to high levels of mobile population mixing based on an assessment using the CDC’s Population Connectivity Across Borders tool^17^: 12 HCFs, 15 points of entry or police checkpoints (POE), 12 guesthouses, 10 markets, seven schools, and four places of worship.

### Data collection.

Assessment visits at each location were performed in January–April 2021. The HCFs were assessed using a CDC tool focused on water, sanitation, and hygiene (WASH),^18^ modified to focus on hand hygiene and water access. This tool was adapted for use in community settings (see Supplemental Appendices A and B).

In HCFs, the tool assessed clinical services offered, the presence of ABHR and handwashing supplies, water sources, and the presence of ABHR and handwashing supplies in patient care areas (Supplemental Appendix A). For community settings, questions covered facility size, location, number of patrons, presence of ABHR and handwashing supplies, and water sources (Supplemental Appendix B). Responses were entered into SurveyCTO (version 2.71; Dobility, Inc., Cambridge, MA). In community settings, schematic drawings were created in consultation with location managers and included entrances/exits, latrines, eating areas, congregation areas, food preparation areas, current handwashing stations, and ABHR (Supplemental Appendix C).

Direct observations of hand hygiene by HCWs and patrons in healthcare and community settings were also performed (Supplemental Appendices D–F). In both settings, appropriate hand hygiene was defined as the use of soap and water for handwashing or the use of ABHR. Use of water alone for handwashing and, for healthcare providers, the use of gloves without prior handwashing or ABHR use were not considered appropriate hand hygiene.

In HCFs, enumerators observed hand hygiene practices of up to five HCWs before and after patient interaction.^12^ Enumerators recorded the type of HCW, whether the interaction was with a new or returning to an existing patient, whether the procedure performed was invasive or noninvasive, the availability of hand hygiene materials, and whether hand hygiene was performed before and after the patient interaction. Contact with the surrounding environment (e.g., touching shared medical equipment) constituted a break in a patient encounter. Enumerators followed a HCW for up to five patient interactions or 1.5 hours (whichever came first). Enumerators notified HCWs that they were observing medical procedures but did not specify that they were observing hand hygiene. HCW observations were recorded on paper (Supplemental Appendix D) and then entered into SurveyCTO.

In community settings, enumerators observed patron hand hygiene practices at entrances, exits, and after latrine use for 30 minutes or 20 observations, whichever came first. To reduce reactivity, enumerators positioned themselves as far away as possible while being able to observe both the observation area and the hand hygiene station.^19^ Sex and approximate age (estimated by the observer as child (roughly < 12–13 years old), adolescent (roughly 12–13 to 18 years old), or adult (roughly > 18 years old) of patrons were recorded as well as the hand hygiene materials available and the materials used (if any) by patrons. Observations of patients and visitors at entrances, exits, and latrines of HCFs were analyzed with those of other community locations. Patron observations were entered directly into SurveyCTO. Paper forms were used if the online platform or electronic device used for recording was not functioning at the time of data collection (Supplemental Appendices E and F).

### Analysis and modeling.

Descriptive analyses and regression models for access to and use of hand hygiene were conducted in SAS version 9.4 (SAS Institute, Cary, NC). For all models, we identified interactions of interest by examining the cross-tabulations of factors with plausible associations with outcomes. We then assessed these potential interactions using tests of generalized score statistics (for generalized estimating equations [GEE] models) or likelihood ratio tests (other models). We used an α of 0.05 to determine statistical significance. We identified characteristics associated with outcomes of interest by estimating odds ratios (ORs) for individual binary outcomes (hand hygiene access in a given patient care area, its use by HCWs, its use by patrons) or by estimating prevalence ratios (PR) for count-based outcomes (number of hand hygiene facilities in a given community location).

### Hand hygiene access: HCFs.

The odds of HCF rooms having hand hygiene materials available were modeled using logistic regression with GEE to account for clustering by HCF. We adjusted ad hoc for the type of room (inpatient rooms, laboratory or staff rooms, and outpatient rooms), source of drinking water for the HCF, and HCF level (Health Center III or IV, hospitals).

### Hand hygiene access: community settings.

The number of entrances/exits or congregation areas with hand hygiene materials present out of total entrances/exits and congregation areas in a community location was estimated using modified Poisson regression, adjusted for the district, location type, access to a water source, and the type of water source.

### Hand hygiene use: HCFs.

The odds of a HCW performing hand hygiene were modeled using logistic regression with GEE accounting for repeat observations within providers in HCF. These models were adjusted for HCW type, new versus existing patient, invasive or noninvasive procedure, HCF level, and whether the moment for hand hygiene was before or after the patient encounter.

### Hand hygiene use: community setting.

The odds of a community setting patron performing hand hygiene was evaluated using logistic regression, adjusting for the type of location, location of observation (entrance/exit or a latrine), age and sex of the patient, and presence of an attendant enforcing or encouraging hand hygiene.

### Ethics.

Protocols for data collection were reviewed by institutional review boards in Uganda (Infectious Diseases Institute and Uganda National Council for Science and Technology) and at CDC and were determined to be nonresearch (CDC project id: 0900f3eb81d6329d).

## RESULTS

### Hand hygiene access: HCFs.

Of the 12 HCFs assessed, 11 (92%) reported ever having ABHR on site, of which only one (9.1%) reported the amount of ABHR was always sufficient for HCF needs (Table 1). Water was onsite at all HCFs, and the most common water source used for handwashing was piped water (75%). Half of the HCFs reported interruptions to the water supply used for handwashing, four HCFs (33%) reported not having enough water for handwashing in the past 2 weeks. Within HCFs, 64% of inpatient, 71% of outpatient, and 90% of laboratory and staff rooms contained hand hygiene materials. All laboratory, maternity, delivery, duty, staff, or storage/unused rooms, but only 18% of patient care rooms (such as wards), contained at least one type of hand hygiene material (Supplemental Table 1). Handwashing stations were present in 59% of rooms and ABHR in 54% of all rooms (Supplemental Table 2). In all, 73% of all handwashing stations assessed were functional (not broken or in need of repair) and had water and soap. Therefore, 43% of all HCF rooms contained a functional handwashing station with soap and water.

**Table 1:** Hand hygiene availability in healthcare facilities

Facilities	HC III	HC IV	Hospital	All
*N* (%)	5 (42)	4 (33)	3 (25)	12
ABHR supply, *n* (%)				
ABHR present anywhere on site	4 (80)	4 (100)	3 (100)	11 (92)
Amount of ABHR sufficient to meet all needs	1 (25)	0 (0)	0 (0)	1 (9)
Interruptions to receiving ABHR*	1/2 (50)	1 (25)	2 (100)	4 (50)
Water supply, *n* (%)				
Water source				
Piped water	4 (80)	2 (50)	3 (100)	9 (75)
Borehole	1 (20)	1 (25)	0 (0)	2 (17)
Unprotected well	1 (20)	0 (0)	0 (0)	1 (8.3)
The water source on location grounds†	5 (100)	4 (100)	3 (100)	12 (100)
Are there ever water interruptions?	2 (40)	3 (75)	1 (33)	6 (50)
In the past 2 weeks, has there always been enough water for handwashing?	4 (80)	3 (75)	1 (33)	8 (67)
Patient care rooms				
*N* (%)	28 (28)	36 (26)	37 (37)	101 (100)
Any hand hygiene available	21 (75)	39 (68)	30 (81)	76 (75)
Inpatient room	4 (80)	6 (50)	6 (75)	16 (64)
Laboratory or staff room	8 (89)	8 (89)	12 (92)	28 (90)
Outpatient room	9 (64)	25 (69)	12 (75)	32 (71)
ABHR details				
≥ 1 ABHR dispenser present in the room	15 (54)	16 (44)	23 (86)	54 (53)
Handwashing station details				
Handwashing station present	17 (61)	19 (53)	24 (65)	60 (59)
Water present	14 (82)	13 (72)	23 (96)	50 (85)
HWS functional	16 (94)	14 (74)	22 (96)	52 (88)
Soap present	12 (71)	12 (63)	21 (88)	45 (75)
Water is present, HWS functional, and soap present	11 (39)	11 (31)	20 (54)	42 (42)

ABHR = alcohol-based hand rub; HC = health center; HSW = handwashing station.

* Eight healthcare facilities responded to this question.

† For all other sites, water access was ≤ 500 m away.

In adjusted models for access to hand hygiene in HCFs, inpatient (OR = 0.20, 95% CI: 0.08–0.52) and outpatient (OR = 0.25, 95% CI: 0.09–0.68) rooms had lower odds of containing hand hygiene materials compared with laboratory or staff rooms (Table 2).

**Table 2:** Unadjusted and adjusted estimates for hand hygiene resource availability in healthcare facilities by room type, source of drinking water, and healthcare facility type

Parameter		Unadjusted		Adjusted*	
		OR	95% CI	OR	95% CI
Room type	Inpatient room	0.19	0.07–0.55	0.20	0.08–0.52
	Outpatient room	0.26	0.10–0.67	0.25	0.09–0.68
	Laboratory or Staff room	Ref.		Ref.	
Source of drinking water	Not piped†	0.42	0.16–1.11	0.41	0.13–1.30
	Piped water	Ref.		Ref.	
Healthcare facility type	HC III	0.70	0.17–2.90	1.13	0.30–4.25
	HC IV	0.53	0.22–1.30	0.91	0.32–2.62
	Hospital	Ref.		Ref.	

HC = health center; OR = odds ratio; Ref. = reference.

* Adjusted for room type, drinking water source, and healthcare facility type.

† Includes unprotected well or manual borehole.

### Hand hygiene access: community settings.

Across location types, the prevalence of hand hygiene materials at entrances/exits was highest in schools (70%, Table 3) and lowest in markets (22%). Prevalence of hand hygiene materials in congregation areas within community locations (e.g., eating areas) was highest in places of worship (63%) and lowest in guesthouses (32%). ABHR was most frequently reported in schools (86%) and least frequently reported in markets (20%); most locations except POEs reported insufficient ABHR to meet all needs. All markets, half of the schools, and points of entry reported interruptions in their ABHR supply line. The most common water source at places of worship, POEs, and guesthouses were piped water, while schools had mostly piped and manual boreholes. Water sources for markets were more mixed. All places of worship, guesthouses, and schools; 71% of POEs; and 44% of markets had onsite water sources. Although 76% of locations reported ever having water interruptions, 83% reported having enough water for handwashing in the past 2 weeks. Further information on study sites can be found in Supplemental Table 3.

**Table 3:** Hand hygiene materials availability in community locations

Location type	Markets	Place of worship	Guest houses	Schools	Point of entry	All
*N* (%)	10 (22)	4 (8.3)	12 (25)	7 (15)	15 (31)	48
ABHR supply, *n* (%)						
ABHR present anywhere on site	2 (20)	1 (25)	5 (42)	6 (86)	8 (53)	22 (46)
Amount of ABHR sufficient to meet all needs (*N* = 22)	0 (0)	1 (100)	1 (20)	0 (0)	6 (75)	8 (36)
Interruptions to receiving ABHR (*N* = 22)	2 (100)	0 (0)	1 (20)	3 (50)	4 (50)	10 (46)
Water supply, *n* (%)						
Piped water	3 (30)	3 (75)	5 (42)	3 (43)	11(73)	25 (52)
Manual borehole	3 (30)	1 (25)	3 (25)	3 (43)	3 (20)	13 (27)
Protected well	3 (30)	0 (0)	4 (33.3)	0 (0)	0 (0)	7 (14.6)
Unprotected well	0 (0)	0 (0)	0 (0)	0 (0)	0 (0)	0 (0)
Tank	0 (0)	0 (0)	0 (0)	1 (14.3)	0 (0)	1 (2.1)
No water source	1 (10)	0 (0)	0 (0)	0 (0)	1 (6.7)	2 (4.2)
Water source on grounds location*	4 (44)	4 (100)	12 (100)	7 (100)	10 (71)	37 (60)
Are there ever water interruptions?	6 (67)	3 (75)	6 (50)	6 (86)	14 (100)	35 (76)
In the past 2 weeks, has there always been enough water for handwashing?	3 (60)	4 (100)	4 (67)	5 (83)	9 (100)	25 (83)
Hand hygiene availability,† *n* (%)						
All entrances/exits with hand hygiene materials present‡ (*N* = 116)	9 (22)	5 (46)	6 (30)	7 (70)	17 (52)	44 (38)
% congregation areas with hand hygiene materials present (*N* = 131)	9 (35)	5 (63)	6 (32)	20 (37)	6 (24)	46 (35)

ABHR = alcohol-based hand rub.

* For sites without water sources on the grounds, water access was ≤ 500 m away in all cases.

† Hand hygiene availability is defined as the presence of ABHR or a handwashing station.

‡ Of 21 entrances/exits assessed in the healthcare facility, 10 (48%) had hand hygiene materials present.

For hand hygiene availability in community settings (limited to entrances/exits and congregation areas, but not toilets), models adjusted for the district, location type, and water source showed no statistically significant associations, although hand hygiene availability was borderline lower for locations with nonpiped water versus piped water (Table 4). We were not able to test interactions of location type and water source due to small sample size.

**Table 4:** Unadjusted and adjusted estimates for hand hygiene resource availability in community settings

Parameter		Unadjusted		Adjusted*	
		PR	95% CI	PR	95% CI
District	Amuru	0.42	0.24–0.73	0.56	0.30–1.06
	Tororo	Ref.		Ref.	
Location type	POE	0.94	0.47–1.87	0.83	0.47–1.49
	Guesthouse	0.73	0.29–1.84	0.67	0.31–1.46
	Place of worship	1.25	0.61–2.54	0.79	0.41–1.54
	Market	0.64	0.26–1.54	0.67	0.26–1.47
	School	Ref.		Ref.	
Water source	Not piped	0.43	0.23–0.81	0.53	0.27–1.02
	No water	0.47	0.06–3.77	0.58	0.06–5.73
	Piped water	Ref.		Ref.	

POE = point of entry; PR = prevalence ratio; Ref. = reference.

* Adjusted for the district, location type, access to water source, and water source.

### Hand hygiene use: HCFs.

In 442 direct observations of hand hygiene by HCWs (221 before patient encounter, 221 after patient encounter), hand hygiene was performed before 26% of patient encounters and after 50% of patient encounters, most commonly by using ABHR (23% and 34%, respectively), resulting in an overall hand hygiene rate of 38% (Table 5). Doctors performed hand hygiene most often (68%) and nurses and laboratory technicians least often (29% and 30%, respectively). Before patient interaction, HCWs donned new gloves without prior handwashing or ABHR use in 33% of observations. Laboratory technicians had a low rate of hand hygiene at 4% and most frequently donned gloves without handwashing or using ABHR. Gloves were present in the room at 84% of observations, followed by ABHR (71%), and soap and water (59%).

**Table 5:** Overall hand hygiene adherence rates in healthcare facilities by healthcare worker type

Variable		Materials available by hand hygiene observations, *n* (%)	Appropriate hand hygiene performed,* *n* (%)
Before patient contact	Total	221 (50)	57 (26)
	Gloves	186 (84)	72 (33)
	Soap and water	131 (59)	14 (6.3)
	Chlorinated water	0 (0)	0 (0)
	ABHR	157 (71)	51 (23)
	None	0 (0)	92 (42)
After patient contact	Total	221 (50)	110 (50)
	Soap and water	131 (59)	35 (16)
	Chlorinated water	0 (0)	0 (0)
	ABHR	157 (71)	75 (34)
	None	0 (0)	111 (50)
Overall	Any hand hygiene performed	441 (100)	167 (38)
		No. of hand hygiene observations, *n* (%)	Appropriate hand hygiene performed,* *n* (%)
Type of healthcare worker (*n*)	Doctor (5)	40 (9.0)	27 (68)
	Midwife (9)	76 (17)	27 (36)
	Nurse (17)	144 (33)	43 (30)
	Clinical officer (11)	90 (20)	43 (48)
	Laboratory tech (11)	92 (21)	27 (29)†
Type of contact‡	Invasive	154 (35)	46 (30)
	Noninvasive	286 (65)	119 (42)

ABHR = alcohol-based hand rub.

* Appropriate hand hygiene includes the use of soap and water or ABHR (does not include the use of gloves alone).

† Laboratory technician hand hygiene rate is 4.4% before patient interaction.

‡ Two were missing.

In the adjusted model for HCW hand hygiene adherence, doctors had higher odds of performing hand hygiene than nurses (OR = 3.58, 95% CI: 1.15–11.14, Table 6). The odds of an HCW performing hand hygiene were higher for new patients versus returning to interact with the same patient after contact with the surroundings (OR = 2.27, 95% CI: 1.20–4.27). Interactions between contact type (invasive versus noninvasive) and the timing of the patient interaction (before versus after-patient contact) were significant. Compared with after patient contact, the odds of before-patient contact were low for invasive contacts (OR = 0.66, 95% CI: 0.45–0.95) but even lower for noninvasive contacts (OR = 0.03, 95% CI: 0.00–0.20).

**Table 6:** Unadjusted and adjusted estimates for hand hygiene adherence of healthcare workers in healthcare facilities

Parameter			Unadjusted		Adjusted*	
			OR	95% CI	OR	95% CI
Healthcare worker type	Clinical officer		2.15	0.86–5.36	2.30	0.87–6.09
	Doctor		4.88	1.59–14.94	3.58	1.15–11.14
	Laboratory technician		0.98	0.51–1.87	1.07	0.42–2.72
	Midwife		1.29	0.48–3.48	1.39	0.52–3.70
	Nurse		Ref.		Ref.	
New patient	Yes		1.92	1.07–3.47	2.27	1.20–4.27
	No		Ref.		Ref.	
Facility level	Health Center IV		1.12	0.52–2.41	1.20	0.50–2.88
	Hospital		1.81	0.87–3.79	1.81	0.80–4.12
	Health Center III		Ref.		Ref.	
Patient interaction	Invasive contact					
	Before		0.03	0.00–0.20	0.03	0.00–0.20
	After		Ref.		Ref.	
	Noninvasive contact					
	Before		0.69	0.49–0.97	0.66	0.45–0.95
	After		Ref.		Ref.	

OR = odds ratio; Ref. = reference.

* Adjusted for patient interaction, contact type, healthcare worker type, new patient, and facility level.

### Hand hygiene use: community settings.

Direct observations of hand hygiene took place at the entrances/exits, and outside latrines at three guesthouses, eight HCFs, four markets, six points of entry, four schools, and two places of worship; 10 entrances/exits and five latrines had no observations due to either no water or absence of hand hygiene station. Most observations were at HCFs (37%), of male patrons (56%), and at entrances/exits (67%, Table 7). Handwashing stations were most commonly present (79%), followed by both handwashing stations and ABHR (17%), then ABHR only (4%). Hand hygiene was attempted in 30% of observations, but 23% counted as appropriate hand hygiene adherence (use of soap and water or ABHR). Soap and water was the most common hand hygiene method used by patrons (62%). Hand hygiene was attempted most frequently at markets (62%) and least frequently at points of entry (21%). Hand hygiene was performed twice as often after latrine use (48%) than entering or exiting a facility (21%).

**Table 7:** Hand hygiene rates in community settings by patron and location type

Variable		No. of hand hygiene observations, *n* (%)	Hand hygiene performed, *n* (%)
Type of location	Point of entry	119 (18)	25 (21)
	Guesthouse	58 (8.8)	17 (29)
	Place of worship	71 (11)	27 (38)
	Market	68 (10)	42 (62)
	Healthcare facility	242 (37)	53 (22)
	Schools	99 (15)	33 (33)
Area	Entrance/exit	441 (67)	93 (21)
	Latrine	216 (33)	104 (48)
Attendant	Present	213 (32)	60 (28)
	Not present	444 (68)	137 (31)
Patron age*	Child	38 (5.8)	7 (18)
	Adolescent	135 (21)	52 (39)
	Adult	484 (74)	138 (29)
Patron sex	Female	292 (44.)	100 (34)
	Male	365 (56)	97 (27)
Type of hand hygiene present	ABHR dispenser	28 (4.3)	8 (29)
	Handwashing station	520 (79)	36 (33)
	Both ABHR and handwashing station	109 (17)	153 (29)
Type of hand hygiene performed	Plain water only		47 (24)
	Water and soap		123 (62)
	Chlorinated water		1 (0.5)
	ABHR		26 (13)
Time spent performing hand hygiene	< 20 seconds		75 (38)
	≥ 20 seconds		122 (62)

ABHR = alcohol-based hand rub.

* Approximate age was estimated by the observer as child (roughly < 12–13 years old), adolescent (roughly 12–13 to 18 years old), or adult (roughly > 18 years old).

In the adjusted model, children had lower odds of performing hand hygiene than adults (OR = 0.37, 95% CI: 0.15–0.92, Table 8). Patrons at places of worship (OR = 2.82, 95% CI: 1.28–6.20) and markets (OR = 2.19, 95% CI: 1.01–4.40) had higher odds of performing hand hygiene than students in schools (the reference). The odds of patrons performing hand hygiene after using the latrine were 3.39 times higher than at a community entrance or exit (95% CI: 2.08–5.52). Attendants present at a location were not associated with the odds of performing hand hygiene.

**Table 8:** Unadjusted and adjusted estimates for hand hygiene adherence by patrons in community settings at entrances/exits and congregation points

Parameter		Unadjusted		Adjusted*	
		OR	95% CI	OR	95% CI
Age	Child	0.54	0.23–1.25	0.37	0.15–0.92
	Adolescent	1.45	0.98–2.16	0.98	0.59–1.60
	Adult	Ref.		Ref.	
Location type	Point of entry	0.45	0.25–0.81	0.85	0.43–1.67
	Guesthouse	0.70	0.35–1.38	1.41	0.62–3.21
	Place of worship	1.03	0.56–1.91	2.82	1.28–6.20
	Market	2.72	1.46–5.07	2.19	1.01–4.40
	Healthcare facility	0.47	0.29–0.77	0.75	0.43–1.33
	Schools	Ref.		Ref.	
Sex	Female	1.43	1.03–1.99	1.29	0.89–1.87
	Male	Ref.		Ref.	
Attendant	Present	0.98	0.69–1.39	1.41	0.87–2.27
	Absent	Ref.		Ref.	
Area type	Latrine	3.23	2.28–4.56	3.39	2.08–5.52
	Entrance/exit	Ref.		Ref.	

OR = odds ratio; Ref. = reference.

* Adjusted for patron age and sex, location type, presence of an attendant, and area type.

## DISCUSSION

This assessment of hand hygiene access and use in two districts in Uganda with high population mobility suggests that despite the ongoing pandemic and associated increases in awareness of preventive behaviors, appropriate hand hygiene was infrequently performed (< 50% during key moments) in HCFs and community locations. Access to hand hygiene materials in HCFs was lower in the patient care rooms than in laboratories and administrative rooms, suggesting a need for prioritization at the point of care. Hand hygiene practice among HCWs was highest after patient contact, reflecting a need for continued emphasis for HCWs to practice hand hygiene to protect patients in addition to themselves. In community settings, markets had lower access to hand hygiene materials than schools, and locations with piped water had borderline better access to hand hygiene materials compared with nonpiped water, suggesting new areas of prioritizing increased water quantity and availability to enable hand hygiene. Hand hygiene practice was more than twice as likely outside latrines than at entrances/exits, emphasizing the need for consistent messaging on hand hygiene at key moments, particularly at novel key moments that emerged during the COVID-19 pandemic such as entrances and exits.^2^^,^^20^

This assessment is one of the few to compare hand hygiene access and use between both healthcare and community settings.^6^ Critically, 38% of HCWs interacting with patients, 48% of patrons after using a latrine, and 21% of patrons entering and exiting a community setting were observed to practice hand hygiene, representing low adherence despite increases in global and local awareness of hand hygiene as a key preventive measure during the COVID-19 pandemic.^20^ With the COVID-19 pandemic, the importance of mitigation measures—including hand hygiene—in community spaces has grown. With the exception of schools, however, standardized guidance on WASH access in community spaces is absent to date.^3^^,^^21^^,^^22^ HCF and school guidelines^3^^,^^4^ may inform those for other community spaces, but adaptation and evaluation are needed.

In HCFs in this evaluation, access to hand hygiene in patient care rooms may have been lower than in administration and staff rooms due to differential prioritization of limited funds and supplies. Past studies in limited resource HCFs have also more frequently found hand hygiene materials in staff areas than inpatient or visitor areas,^23^ given limited funds to supply, equip, and maintain handwashing stations in HCFs are frequently reported,^24^^,^^25^ such as too few staff to refill handwashing stations.^26^ For ABHR, staff or facility administrators may limit access in areas with patient or visitor traffic (including patient care areas) to ensure sustained ABHR access to staff, especially in high-use situations such as pandemics. Given the quicker ease of use and reduced burden on water supply, increased access to ABHR can improve overall hand hygiene adherence.^27^

Healthcare workers may have had better hand hygiene adherence after patient contact, compared with before, because they felt their own hands were “dirty” after patient contact and may have been motivated to protect themselves from infection or illness.^28^ This inherent feeling of perceived (or actual) contamination can be a stronger motivator to perform hand hygiene than the elected practice of performing hand hygiene on hands that “feel” clean before patient contact.^29^ Similarly, protection of self—more so than patient—may be an important motivator: for example, past research in Arua, Uganda indicated that previous nosocomial infection in an HCW was associated with higher levels of hand hygiene.^30^ Training and reminders on protecting patients for HCWs, in addition to increased access to ABHR, may be needed to improve hand hygiene adherence in HCF.

The COVID-19 pandemic has emphasized the need for hand hygiene in community settings, such as markets, alongside locations with long-standing guidance for WASH access (e.g., schools).^2^ Markets, in particular, are key community locations with close interactions between patrons and vendors. Hand hygiene needs in community settings reinforce the need for concomitant water sources meeting safely managed criteria within the drinking water ladder. Hand hygiene materials were available in a few entrances/exits or congregation areas (38% and 35%, respectively). Hand hygiene can only take place when supplies are available. In addition to hand hygiene stations themselves, water that is onsite and available when needed may be an applicable initial benchmark in these settings.^4^^,^^31^^,^^32^ Even among households, which have less water demand than community locations, multiple sources are sometimes required to maintain sufficient water quantity for needs beyond drinking^33^^,^^34^; additional demands for water for hygiene in community spaces present challenges to consistent handwashing in LMIC settings.^35^ Water-efficient solutions for handwashing stations, such as soap and water designs that minimize wastage while not in use,^36^^–^^38^ may also reduce demands on water supply. Complementary use of ABHR can also increase access with a smaller footprint than handwashing stations, although ABHR should be prioritized where hands are least likely to be visibly dirty and with consistent messaging to reinforce appropriate use.^39^ Local production of ABHR may enable more affordable, consistent access in community settings, although supply chain models are currently undefined, as are roles and guidance for management.^6^ Community settings may require managers or influencers to ensure hand hygiene materials are consistently available and used, similar to the role played by infection prevention and control focal persons in HCFs.^9^^,^^40^^,^^41^

Higher rates of hand hygiene after latrine use than at entrances or exits emphasizes the need for sustained, consistent messaging tying hand hygiene to key moments, particularly to novel key moments during a pandemic. Long-standing guidance emphasizes hand hygiene at multiple key moments in community settings (including after toileting, around food preparation and eating, and after touching animals, or sneezing, coughing, or blowing one’s nose, among others), many of which are times for handwashing specifically.^22^^,^^42^ Hand hygiene—and handwashing specifically—after using the toilet has been most commonly emphasized in WASH initiatives. Hand hygiene at entrances and exits to community spaces is a COVID-19 pandemic-related guidance^43^^,^^44^ that may improve access to hand hygiene facilities but may not always be tied to an existing key moment (and consequently, existing habits) or may be subject to social pressures that make it more difficult to form habits and yield lower adherence.^22^ If these locations are to be considered key moments for hand hygiene beyond the pandemic, user-centered approaches to ensuring coherent and more generalizable evidence-based messaging on these moments may be necessary.^45^

This assessment had several limitations. First, the generalizability of the two districts analyzed should be considered as other districts in Uganda, and in other LMICs, may have had different supplies and rates of hand hygiene. Although study teams were familiar with the methodology of direct observation of hand hygiene from previous assessments,^46^ trainings of enumerators could only be given remotely during the pandemic. Despite messaging to HCWs that observations were about medical practices generally and not hand hygiene specifically, HCWs could have changed practices during observations due to the Hawthorne effect.^47^ Additionally, frequent COVID-19 awareness campaigns during this time period could have resulted in greater attention to hand hygiene during this period and potentially overestimated rates even though rates were already relatively low.

Although this assessment revealed basic hand hygiene material access and use, further information on the supply chain of ABHR (e.g., factors preventing a sufficient amount from being available^7^^,^^8^), barriers to use (e.g., concern among religious groups for ABHR containing alcohol^48^), and current educational resources and gaps (e.g., emphasizing that donning gloves without first using ABHR or soap and water does not constitute appropriate hand hygiene^49^) are needed.

Even during the global COVID-19 pandemic, hand hygiene rates were low in both community and healthcare settings. In HCFs, increased access to hand hygiene materials in patient care areas and emphasis on appropriate hand hygiene before patient interactions are needed. In community settings, emphasis on hand hygiene at key moments and guidance and messaging for new community locations requiring hand hygiene are actionable next steps to improve access to and use of hand hygiene across LMICs.

## Supplemental Materials


Supplemental materials
