# Supplementary material for: Access to and Use of Hand Hygiene Resources during the COVID-19 Pandemic in Two Districts in Uganda, January–April 2021
Source: Am J Trop Med Hyg. 2023 Aug 28;109(4):881–9. doi: 10.4269/ajtmh.23-0031 (PMC10551090; doi:10.4269/ajtmh.23-0031)
Supplement: Supplementary file 1 [file tpmd230031.SD1.pdf]

Supplemental Material

Supplemental Table 1. Hand hygiene material availability by detailed room type in healthcare facilities.

Room type	Number of rooms with HH materials present	Total number of rooms
Administrative	4 (80)	5
Care room	2 (18)	11
Consultation room	15 (83)	18
Delivery room	4 (100)	4
Dispensary	4 (80)	5
Duty room	5 (100)	5
Injection room	1 (50)	2
Laboratory	11 (100)	11
Maternity room	8 (100)	8
Other	6 (67)	9
Prenatal room	1 (100)	1
Resting room	0 (0)	1
Staff room	3 (100)	3
Storage or unused	1 (100)	1
Vaccination room	3 (60)	5
Waiting area	8 (67)	12
**Total**	**76 (75)**	**101**

Supplemental Table 2. Type of hand hygiene materials in healthcare facilities by the room.

Hand hygiene materials present	Rooms, n (%)
**ABHR**	**54 (53)**
Spray bottle	56 (97)
Pump top bottle	1 (2)
Push-style	1 (2)

**Handwashing station**	**60 (59)**
Open the container with tap	11 (18)
Covered container with tap	5 (8)
Sink with tap	43 (72)
Other	1 (2)

Supplemental Table 3. Patrons and staff by location type.

Location type	Number of locations	Mean number of daily patrons (range)	Mean number of daily staff (range)
**Markets**	10	565 (30 – 1000)	*Staff*: 22 (0 – 68)
			Vendors: 392 (20 – 2000)
**Places of worship**	4	540 (160 – 1500)	12 (6-29)
**Guesthouses**	12	27 (8 – 60)	16 (4 – 76)
**Schools**	7	468 (43 – 1000)	68 (13 – 158)
**POEs**	15	1339 (0 – 6000)	59 (9 – 296)
**Healthcare facilities**	12	*Outpatient (monthly)*: 864 (100 – 2000)	*Clinical*: 29 (8 – 100)
		In patient (monthly): 129 (15 – 400)	*Non-clinical*: 12 (0 – 150)

Appendix A. Healthcare Facilities Baseline WASH Assessment Tool.

**Assessment date: ___/___/_____**				
A1	Interviewer:			
A2	Name of District:			
A3	What type of health facility is this?	1	HC II	
		2	HC III	
		3	HC IV	
A4	Name of health facility:			
A5	Position of interviewee:			
A6	Read paper consent form			
A7	Are you willing to participate?	0	No	*If no, survey ends*
		1	Yes	
A8	Administrative status of health facility:	1	Public	
		2	Private	
**Clinical Services**<br>We would like to get an idea of the available clinical services at this health facility.				
B1	Number of average monthly outpatient consultations:			
B2	Number of average monthly admissions (including deliveries):			
**Health Center Staff**<br>Now, we would like to ask about the number of staff at this facility.				
B3a	Number of clinical staff:			
B3b	Number of non-clinical staff:			
**ABHR Supplies**<br>Now, I want to ask you questions about alcohol handrub supplies at your Health Center.				
C1	Does this facility ever have an alcohol-based handrub	0	No	*If no, skip*

	on site?	1	Yes	*to D1*
C2	Where does the alcohol-based handrub come from? (Select all that apply)	1	Government	
		2	Private organization (NGO, nonprofit, etc.)	
		3	Facility makes it themselves	
		4	IDI brand ABHR	
		88	Other	
C2a	Specify other			*Ask if C2=88*
C3	What recipe is the facility using to make alcohol-based handrub?	1	WHO formula with ethanol (ethanol 80%, glycerol 1.45%, hydrogen peroxide 0.125%)	*Ask if C2=3*
		2	WHO formula with isopropyl alcohol (isopropyl alcohol 75%, glycerol 1.45 %, hydrogen peroxide 0.125%)	
		88	Other	
		99	Do not know	
C3a	Specify other			*Ask if C3=88*
C4	*Test alcohol concentration using an alcoholmeter*			*Ask if C2=3*
C5	Is the amount of handrub always sufficient for all needs?	0	No	*If yes, skip to C7*
		1	Yes	
C6	What are the reasons there is not always a sufficient amount of ABHR? (Select all that apply)	1	Not available for purchase / not in stock at the central store	
		2	Health facilities cannot afford	
		3	Stock runs out before the next shipment arr.lvess	
		4	Delivery delayed	

		88	Other	
C6a	Specify other			*Ask if C6=88*
C7	Does this facility have a formal supply line of alcohol-based handrub?	0	No	*If no, skip to C10*
		1	Yes	
C8	Are there ever any interruptions to supply?	0	No	*If no, skip to C10*
		1	Yes	
C9	What are the reasons for interruption in the alcohol-based handrub supply line?	1	ABHR ingredient shortages	
		2	Dispenser shortages	
		3	Payment issues	
		4	Delivery issues	
		5	Other	
C10	Who has access to ABHR at this health facility? *(Select all that apply)*	1	Providers	
		2	Support staff	
		3	Patients	
		4	Family members/visitors	
		88	Others	
C10a	Specify other			*Ask if C10=88*
C11	Do providers regularly carry alcohol handrub on their person as they work? If so, most or some?	2	Yes, most providers regularly carry alcohol hand rub	*Do not ask if C1=1 is not selected*<br><br>*If no, skip to D1*
		1	Yes, some providers regularly carry alcohol handrub	
		0	No	
C12	Where do providers get the handrub that they carry on their person? *(Select all that apply)*	1	They purchase it themselves	
		2	From the health facility's	

			stock	
		88	Other	
C12a	Specify other			*Ask if C12=88*
**Water supply**<br><br>Now, I would like to ask you questions about the water sources at this facility.				
D1	What is the water source used for handwashing at this health facility today?	1	Piped water	
		2	Borehole (has a small diameter and was dug with a machine)	
		Wh 3	Protected dug well (well with a large diameter and a cover)	
		4	Unprotected dug well (well with a large diameter and no cover)	
		5	Rainwater harvest tank	
		6	Tanker truck	
		7	Unprotected spring	
		8	Protected spring (catchment area and spring head are protected from contamination)	
		9	Surface water (river, stream, lake, etc.)	
		99	Do not know	
		88	Other:	
D1a	Specify other			*Ask if D1=88*
D2	What is the original source of the piped water?	1	Municipal system	*Ask if D1=1*
		2	Mechanized borehole	
		88	Other	

		99	Do not know	
D2a	Specify other			*Ask if D2=88*
D3	Where do you access this water source – within the grounds of the health facility, within 500 meters of the health facility, or more than 500 meters from the health facility?<br><br>*Note: this can be from a tap or from the source itself*	2	On the grounds of the health facility	
		1	Within 500 meters of the health facility	
		0	More than 500 meters from the health facility	
D4	Are there ever interruptions to the water supply used for handwashing?	1	Yes	*If no, skip to D6*
		0	No	
D5	During the last calendar year (2019), during how many months of the year was water provision disrupted or NOT available? *(Number)*<br><br>*Note: these do not have to be consecutive months*			
D6	In the past two weeks, has there been enough water at the health facility for handwashing?	1	Yes	
		0	No	

**Additional observations**				**Skip patterns**
F1	How many entrances or exits are present at this facility? *(Number)*			
F2	How many entrances or exits have a handwashing station with soap and water or ABHR present? *(Number)*			

Appendix B. Community Locations Baseline WASH Assessment.

**Assessment date: ___/___/_____**				
A1	Interviewer:			
A2	Name of the interviewee(s) ____________ :			
A3	Position of interviewee:			
A4	Read paper consent form			
A5	Are you willing to participate?	0	No	*If no, survey ends*
		1	Yes	
A6	What type of location is this?	1	POE	
		2	School	
		3	Lodging	
		4	Church	
		5	Market	
		88	Other	
A7	Specify other			*Ask if B6=88*
A6	What is the name of this location?			
A7	What are the GPS coordinates of this location?	Latitude:		
		Longitude:		

**For Markets ONLY**<br><br>Now, we would like to ask about the number of staff/vendors/customers at this facility. We are interested in the average number of people on a typical day, not just how many you think are here today.	

B1	Number of Market health officers/workers/screeners			
B2	Number of security staff (Askari)			
B3	Number of cleaning staff			
B4	Number of URA officials (Tax Collectors)			
B5	Number of other paid or volunteer staff			*describe "other" in B8 if B7 is more than zero*
B6	Please describe "other" staff			
B7	On average, how many vendors come to this market per market day?			
B8	On average, how many customers come to this market per market day?			
B9	Are there any months of the year where there are more vendors and customers than other months (including seasonal or special events)?	0	No	
		1	Yes	
B10	Which months attract more vendors and customers than other months? *Select all that apply.*	1	January	Ask if B9 = 1
		2	February	
		3	March	
		4	April	
		5	May	
		6	June	
		7	July	
		8	August	
		9	September	
		10	October	
		11	November	
		12	December	
B11	In general, what is the most common method of transport that vendors/customers use to come to	1		On foot

	the market? (check only one)		
		2	By car/taxi
		3	By commercial truck
		4	By motorcycle/ boda-boda
		88	Other
B11a	Specify other		*Ask if B11=88*

**For Churches ONLY**<br><br>Now, we would like to ask about the number of staff/parishioners at this Church. We are interested in the average number of people on a typical day, not just how many you think are here today.			
B1	Number of Church leaders		
B2	Number of security staff (guards/Askari)		
B3	Number of cleaning staff		
B4	Number of screeners		
B5	Number of police officers		
B6	Number of other paid or volunteer staff		
B7	Please describe "other" staff		*describe "other" in B8 if B7 is more than zero*
B8	On average, how many worshippers attend Church services per day?		
B9	In general, how do worshippers travel to and from the Church mostly on foot/privately, or in communal vehicles (bus, large taxi, etc.)	1	Privately
		2	Communally

For Guesthouses ONLY<br><br>Now, we would like to ask about the number of staff/guests at this guesthouse. We are interested in the average number of people on a typical day, not just how many you think are here today.				
B1	Number of workers at this guesthouse			
B2	Number of security staff, if any			
B3	Number of cleaning staff			
B4	Number of other paid or volunteer staff (comment section in an electronic tool for further categorization)			
B5	On average, how many travelers/clients access this lodge per day?			
B6	In general, how do travelers/clients come to this guesthouse (check all that apply)	1	On foot	
		2	By car/taxi	
		3	By commercial truck	
		4	By motorcycle/ boda-boda	
		88	Other	
B6a	Specify other			*Ask if B6=88*

For Schools ONLY<br><br>Now, we would like to ask about the number of staff/pupils at this school. We are interested in the average number of people on a typical day, not just how many you think are here today.			
B1	Number of school administrators		
B2	Number of teaching staff		
B3	Number of cleaning staff		
B4	Number of school health care workers		
B5	Number of security officers		

B6	Number of school cooks		
B7	Number of cleaners		
B8	Number of other paid or volunteer staff		*Ask if B8=88*
B8a	Please describe "other" staff		
B9	On average, how many students/pupils attend this school daily?		
B10	How many are;<br>a) boarders<br>b) day scholars		
B11	In general, what is the most common way that the day school students travel to school?	1	On foot
		2	By car/taxi
		3	By motorcycle/ boda-boda
		88	Other
B11a	Specify other		*Ask if B11 = 88*

**For POE ONLY:**<br>Now, we would like to ask about the number of staff at this location. We are interested in the average number of people on a typical day, not just how many you think are here today.			
B1	Number of POE health officers/workers/screeners		
B2	Number of security staff (DISO and BISO)		
B3	Number of cleaning paid or volunteer staff		
B4	Number of customs/immigration staff		
B5	Number of police officers		
B6	Number of URA officials		
B7	Number of other staff (comment section in an electronic tool for further categorization)		

B8	On average, how many travelers pass through this POE (in either direction) per day?			
B9	In general, how do travelers pass through the POE (check all that apply)	1	On foot	
		2	By car/taxi	
		3	By commercial truck	
		4	By motorcycle/ boda-boda	
		88	Other	
B10	Specify other			*Ask if B9=88*
B11	Typically, what is the most common way that travelers pass through the POE (check only once)	1	On foot	
		2	By car/taxi	
		3	By commercial truck	
		4	By motorcycle/ boda-boda	
		88	Other	
B11a	Specify other			*Ask if B11= 88*

C1	In general, is there at least one usable handwashing station present at this location?	1	Yes	*If no, skip to C3*
		2	No	
C2	What types of hand washing stations are typically present for anyone to use? *Select all that apply.*	1	Handwashing stations with soap and water	
		2	Handwashing stations with chlorinated water	
		88	Other	
C3	In general, does this facility have alcohol-based hand rub on site?	0	No	*If no, skip to D1*
		1	Yes	
C4	How many entrances or exits are present at this			Answer C4a if the answer

	location? *(Number)*			is zero to C4
C4a	Explain why there are no formal entrances or exits			
C5	How many entrances or exits typically have a functional handwashing station with soap and water/chlorine or ABHR present? *(Number)* ***Assessor to verify***			
C6	How many areas are there where people (classrooms, cooking areas, cafeterias, large offices)?			*If no, skip to C7*
C6a	How many of these areas (lobby, dining, other) typically have a functional handwashing station with soap and water, chlorinated water, or ABHR present? *(Number)* ***Assessor to verify***			
C7	In general, where does the alcohol-based hand rub come from? (Select all that apply)	1	Government	
		2	Private organization (NGO, nonprofit, etc.)	
		3	Facility makes it themselves	
		88	Other	
C7a	Specify other			*Ask if C7=88*
	What amount of ABHR is received in liters per month?			
C8	In the last 6 months, has the amount of hand rub always been enough for all needs?	0	No	*If yes, skip to C10*
		1	Yes	
		99	I am not sure	
C9	In general, what are the reasons there is not always enough ABHR? (Select all that apply)	1	Not available for purchase / not in stock at the central store	
		2	Cannot afford	
		3	Stock runs out before the next shipment arrives	
		4	Delivery delayed	
		5	Donated periodically or sporadically	

		88	Other	
C9a	Specify other			*Ask if C4=88*
C10	Are there ever any interruptions to the alcohol hand rub supply?	0	No	*If no, skip to C11*
		1	Yes	
		99	I am not sure	
C10a	If Yes, how long was the LAST interruption (in days)?			
C11	What are the usual reasons for interruption in the alcohol-based hand rub supply line?	1	ABHR ingredient shortages	
		2	Dispenser shortages	
		3	Payment issues	
		4	Delivery issues	
		5	Other	
C12	Does this location have a policy that everyone entering the premises must perform hand hygiene?	0	No	
		1	Yes	
C13	Who usually has access to (is able to use) ABHR at this location? *(Select all that apply)*	1	Health officer/workers	
		2	Entry screeners	
		3	URA/Customs/Taxers	
		4	Police	
		5	Travelers	
		6	Truck drivers	
		7	School directors or administrators	
		8	Teachers	
		9	Security officers	
		10	Student/pupils	
		11	Janitors/Cleaners	
		12	Guesthouse staff	
		13	Visitors, attendants or	

			customers	
		14	Vendors	
		15	Church leaders	
		16	VIP	
		17	Lodgers	
		88	Others	
C13a	Specify other			*Ask if C12 = 88*
C14	Do staff regularly carry alcohol hand rub on their person as they work? If so, most or some? ***(Assessor to verify by observation)***	2	Yes, most regularly carry alcohol hand rub	*If no, skip to D1*
		1	Yes, some regularly carry alcohol hand rub	
		0	No	
C15	Where do the staff usually get the hand rub that they carry on their person? *(Select all that apply)*	1	They purchase it themselves	
		2	From the location's stock/supplies	
		88	Other	
C15a	Specify other			*Ask if C12=88*
**ALL locations: Water supply**<br><br>Now, I would like to ask you questions about the water sources at this location				
D1	What is the usual water source used for handwashing at this location?	1	Piped water	*Ask if C4=1*
		2	Borehole (has a small diameter and was dug with a machine)	
		3	Protected dug well (well with a large diameter and a cover)	
		4	Unprotected dug well (well with a large	

			diameter and no cover)	
		5	Rainwater harvest tank	
		6	Tanker truck	
		7	Unprotected spring	
		8	Protected spring (catchment area and spring head are protected from contamination)	
		9	Surface water (river, stream, lake, etc.)	
		88	Other:	
D1a	Specify other			*Ask if D1=88*
D2	What is the original source of the piped water?	1	Municipal system	*Ask if D1=1*
		2	Mechanized borehole	
		88	Other	
		99	Do not know	
D2a	Specify other			*Ask if D2=88*
D3	Where do you typically access this water source – within the grounds, within 500 meters, or more than 500 meters from this location?<br><br>*Note: this can be from a tap or from the source itself*	2	On the grounds of this location	
		1	Within 500 meters of this location	
		0	More than 500 meters from this location	
D4	Are there ever interruptions to the water supply used for handwashing?	1	Yes	*If no, skip to D6*
		0	No	
D5	During the last calendar year (2019), during how many months of the year was water provision disrupted or NOT available for one or more days? *(Number)*			

	Note: these do not have to be consecutive months			
D5a	What are the usual reasons for the water supply interruption at this location?	1	Delay in bill payments	
		1	Mechanical breakdown	
		2	Low water table	
		3	Locked / restricted access	
		4	Power outage	
		5	Seasonal availability	
		6	Inability to pay / too expensive	
		7	Cuts to water system (rationing)	
		88	Others (specify)	
D6	In the past two weeks, has there always been enough water at this location for handwashing?	1	Yes	
		0	No	

Appendix C. Location schematic instructions.

**ALL locations: Location schematic (drawing of the location that shows key areas)**<br>Now, I would like to ask you to draw the schematic of this location, identifying where hand hygiene materials are already present, and places where hand hygiene materials would be most useful<br>**** Facilitator – include the name of the location, district name, and GPS coordinates on the drawing. Take a photograph of the schematic and upload to the ODK form for this location. IF permission is given, include photographs of key places in this location where hand hygiene materials are already, or could be, placed.*		
E1	Please draw, to the best of your ability, your location. Please include and label the following key areas:	
	Entrances and exits	*Label as E*
	Restrooms	*Label as R*
	Places where people eat	*Label as D*
	Places where people congregate, such as lobbies, bus stops, taxi stands, playing areas, etc.	*Label as C*
	Places where food is prepared	*Label as F*
	Places where handwashing stations are typically present	*Label as H*
	Places where alcohol-based hand rub dispensers are typically present	*Label as A*
E2	On the map, please note any additional locations where you think hand hygiene materials should be placed. Consider the needs of both staff and visitors. Also note that ABHR is not enough after using the latrines and that hand washing stations are recommended by latrines.	
	Where do you think hand washing stations would be most useful?	*Label as XH*
	Where do you think ABHR stations would be most useful	*Label as XA*

	Where do you think wall mounted ABHR dispensers would be most useful?	*Label as XW*

Appendix D. Healthcare workers hand hygiene observations tool.

Observer name:____________________ **Hand Hygiene Observation Tool** HCF name:____________________

Date (mm/dd/yy):____________________ Sub-County:____________________

A. HCW #	B. Type of health worker					C. Obs. start time	D. Contact number	D.1. new patient (circle one)	E. Hand hygiene materials present (select all that apply)						E.1. Contact type		F. Hand hygiene BEFORE patient contact (select all that apply)					G. Hand hygiene AFTER patient contact (select all that apply)				H. Obs. end time
	Doctor	Midwife	Nurse	Clinical	Lab				Gloves	Water and soap	Chlorinated water	Water only	ABHR	None	Invasive	Non-invasive	New gloves	Handwashing: soap	Handwashing: chlorinated water (no	ABHR	None	Handwashing: soap	Handwashing: chlorinated water (no	ABHR	None	
							1	Y N	□	□	□	□	□	□	□	□	□	□	□	□	□	□	□	□	□	
							2	Y N	□	□	□	□	□	□	□	□	□	□	□	□	□	□	□	□	□	
1	□	□	□	□	□	__:__	3	Y N	□	□	□	□	□	□	□	□	□	□	□	□	□	□	□	□	□	__:__
							4	Y N	□	□	□	□	□	□	□	□	□	□	□	□	□	□	□	□	□	
							5	Y N	□	□	□	□	□	□	□	□	□	□	□	□	□	□	□	□	□	
							1	Y N	□	□	□	□	□	□	□	□	□	□	□	□	□	□	□	□	□	
							2	Y N	□	□	□	□	□	□	□	□	□	□	□	□	□	□	□	□	□	
2	□	□	□	□	□	__:__	3	Y N	□	□	□	□	□	□	□	□	□	□	□	□	□	□	□	□	□	__:__
							4	Y N	□	□	□	□	□	□	□	□	□	□	□	□	□	□	□	□	□	
							5	Y N	□	□	□	□	□	□	□	□	□	□	□	□	□	□	□	□	□	
							1	Y N	□	□	□	□	□	□	□	□	□	□	□	□	□	□	□	□	□	
							2	Y N	□	□	□	□	□	□	□	□	□	□	□	□	□	□	□	□	□	
3	□	□	□	□	□	__:__	3	Y N	□	□	□	□	□	□	□	□	□	□	□	□	□	□	□	□	□	__:__
							4	Y N	□	□	□	□	□	□	□	□	□	□	□	□	□	□	□	□	□	
							5	Y N	□	□	□	□	□	□	□	□	□	□	□	□	□	□	□	□	□	
							1	Y N	□	□	□	□	□	□	□	□	□	□	□	□	□	□	□	□	□	
							2	Y N	□	□	□	□	□	□	□	□	□	□	□	□	□	□	□	□	□	
4	□	□	□	□	□	__:__	3	Y N	□	□	□	□	□	□	□	□	□	□	□	□	□	□	□	□	□	__:__
							4	Y N	□	□	□	□	□	□	□	□	□	□	□	□	□	□	□	□	□	
							5	Y N	□	□	□	□	□	□	□	□	□	□	□	□	□	□	□	□	□	
							1	Y N	□	□	□	□	□	□	□	□	□	□	□	□	□	□	□	□	□	
							2	Y N	□	□	□	□	□	□	□	□	□	□	□	□	□	□	□	□	□	
5	□	□	□	□	□	__:__	3	Y N	□	□	□	□	□	□	□	□	□	□	□	□	□	□	□	□	□	__:__
							4	Y N	□	□	□	□	□	□	□	□	□	□	□	□	□	□	□	□	□	
							5	Y N	□	□	□	□	□	□	□	□	□	□	□	□	□	□	□	□	□	

Appendix E. Community entrances and exits hand hygiene observations tool.

Hand Hygiene Observation Tool - ENTRANCE / EXITS of Public Places

1. Observer name:__________________ 2. Date (mm/dd/yy):__________________
3. Site name:__________________ 4. Start time: ___ : ___ 5. End time: ___ : ___
6. Type of hand hygiene station present at entrance/exit (select all that apply):
□ Handwashing station (HS) with chlorinated water □ HS with soap and water □ HS with plain water only □ HS with no water * □ ABHR dispenser
7a. Are any IEC materials present? □ Yes □ No 7b. If yes, describe: __________________
8. Is an HW attendant present at entrance/exit □ Yes □ No
*If only a HW station is present, but has no water, do not observe more than 5-10 minutes and skip D and E

A. Person number	B. Did they enter or exit? (circle one)	C. Did they use (or try to use) the hand hygiene station? (circle one)	D. Type of hand hygiene performed (select all that apply)				E. Length of time that hand hygiene is performed	F. Sex	G. Age (in years)
			Handwashing: water only	Handwashing: chlorinated	Handwashing: water and soap	ABHR			
1	Enter / Exit	Yes / No	□	□	□	□	□ < 20 seconds □ ≥ 20 seconds	□ M □ F	□ Child □ Juvenile □ Adult
2	Enter / Exit	Yes / No	□	□	□	□	□ < 20 seconds □ ≥ 20 seconds	□ M □ F	□ Child □ Juvenile □ Adult
3	Enter / Exit	Yes / No	□	□	□	□	□ < 20 seconds □ ≥ 20 seconds	□ M □ F	□ Child □ Juvenile □ Adult
4	Enter / Exit	Yes / No	□	□	□	□	□ < 20 seconds □ ≥ 20 seconds	□ M □ F	□ Child □ Juvenile □ Adult
5	Enter / Exit	Yes / No	□	□	□	□	□ < 20 seconds □ ≥ 20 seconds	□ M □ F	□ Child □ Juvenile □ Adult
6	Enter / Exit	Yes / No	□	□	□	□	□ < 20 seconds □ ≥ 20 seconds	□ M □ F	□ Child □ Juvenile □ Adult
7	Enter / Exit	Yes / No	□	□	□	□	□ < 20 seconds □ ≥ 20 seconds	□ M □ F	□ Child □ Juvenile □ Adult
8	Enter / Exit	Yes / No	□	□	□	□	□ < 20 seconds □ ≥ 20 seconds	□ M □ F	□ Child □ Juvenile □ Adult
9	Enter / Exit	Yes / No	□	□	□	□	□ < 20 seconds □ ≥ 20 seconds	□ M □ F	□ Child □ Juvenile □ Adult
10	Enter / Exit	Yes / No	□	□	□	□	□ < 20 seconds □ ≥ 20 seconds	□ M □ F	□ Child □ Juvenile □ Adult
11	Enter / Exit	Yes / No	□	□	□	□	□ < 20 seconds □ ≥ 20 seconds	□ M □ F	□ Child □ Juvenile □ Adult
12	Enter / Exit	Yes / No	□	□	□	□	□ < 20 seconds □ ≥ 20 seconds	□ M □ F	□ Child □ Juvenile □ Adult
13	Enter / Exit	Yes / No	□	□	□	□	□ < 20 seconds □ ≥ 20 seconds	□ M □ F	□ Child □ Juvenile □ Adult
14	Enter / Exit	Yes / No	□	□	□	□	□ < 20 seconds □ ≥ 20 seconds	□ M □ F	□ Child □ Juvenile □ Adult
15	Enter / Exit	Yes / No	□	□	□	□	□ < 20 seconds □ ≥ 20 seconds	□ M □ F	□ Child □ Juvenile □ Adult
16	Enter / Exit	Yes / No	□	□	□	□	□ < 20 seconds □ ≥ 20 seconds	□ M □ F	□ Child □ Juvenile □ Adult
17	Enter / Exit	Yes / No	□	□	□	□	□ < 20 seconds □ ≥ 20 seconds	□ M □ F	□ Child □ Juvenile □ Adult
18	Enter / Exit	Yes / No	□	□	□	□	□ < 20 seconds □ ≥ 20 seconds	□ M □ F	□ Child □ Juvenile □ Adult
19	Enter / Exit	Yes / No	□	□	□	□	□ < 20 seconds □ ≥ 20 seconds	□ M □ F	□ Child □ Juvenile □ Adult
20	Enter / Exit	Yes / No	□	□	□	□	□ < 20 seconds □ ≥ 20 seconds	□ M □ F	□ Child □ Juvenile □ Adult
21	Enter / Exit	Yes / No	□	□	□	□	□ < 20 seconds □ ≥ 20 seconds	□ M □ F	□ Child □ Juvenile □ Adult
22	Enter / Exit	Yes / No	□	□	□	□	□ < 20 seconds □ ≥ 20 seconds	□ M □ F	□ Child □ Juvenile □ Adult
23	Enter / Exit	Yes / No	□	□	□	□	□ < 20 seconds □ ≥ 20 seconds	□ M □ F	□ Child □ Juvenile □ Adult

Appendix F. Community latrines hand hygiene observations tool.

Hand Hygiene Observation Tool - LATRINES of Public Places

1. Observer name:__________________________ 2. Date (mm/dd/yy):________________________

3. Site name:_______________________________ 4. Start time: ___ : ___ 5. End time: ___ : ___

6. Type of hand hygiene station present at the latrine (select all that apply):

□ Handwashing station (HS) with chlorinated water □ HS with soap and water □ HS with plain water only □ HS with no water * □ ABHR dispenser

7a. Are any IEC materials present by the latrine? □ Yes □ No 7b. If yes, describe:

8. Is a HW attendant present at the latrine? □ Yes □ No

*If only a HW station is present, but has no water, do not observe more than 5-10 minutes and skip D and E

A. Person number	C. Did they use the hand hygiene station? (circle one)	D. Type of hand hygiene performed (select all that apply)				E. Length of time that hand hygiene is performed	F. Sex	G. Age (in years)
		Handwashing: water only	Handwashing: chlorinated	Handwashing: water and soap	ABHR			
1	Yes / No	□	□	□	□	□ < 20 seconds □ ≥ 20 seconds	□ M □ F	□ Child □ Juvenile □ Adult
2	Yes / No	□	□	□	□	□ < 20 seconds □ ≥ 20 seconds	□ M □ F	□ Child □ Juvenile □ Adult
3	Yes / No	□	□	□	□	□ < 20 seconds □ ≥ 20 seconds	□ M □ F	□ Child □ Juvenile □ Adult
4	Yes / No	□	□	□	□	□ < 20 seconds □ ≥ 20 seconds	□ M □ F	□ Child □ Juvenile □ Adult
5	Yes / No	□	□	□	□	□ < 20 seconds □ ≥ 20 seconds	□ M □ F	□ Child □ Juvenile □ Adult
6	Yes / No	□	□	□	□	□ < 20 seconds □ ≥ 20 seconds	□ M □ F	□ Child □ Juvenile □ Adult
7	Yes / No	□	□	□	□	□ < 20 seconds □ ≥ 20 seconds	□ M □ F	□ Child □ Juvenile □ Adult
8	Yes / No	□	□	□	□	□ < 20 seconds □ ≥ 20 seconds	□ M □ F	□ Child □ Juvenile □ Adult
9	Yes / No	□	□	□	□	□ < 20 seconds □ ≥ 20 seconds	□ M □ F	□ Child □ Juvenile □ Adult
10	Yes / No	□	□	□	□	□ < 20 seconds □ ≥ 20 seconds	□ M □ F	□ Child □ Juvenile □ Adult
11	Yes / No	□	□	□	□	□ < 20 seconds □ ≥ 20 seconds	□ M □ F	□ Child □ Juvenile □ Adult
12	Yes / No	□	□	□	□	□ < 20 seconds □ ≥ 20 seconds	□ M □ F	□ Child □ Juvenile □ Adult
13	Yes / No	□	□	□	□	□ < 20 seconds □ ≥ 20 seconds	□ M □ F	□ Child □ Juvenile □ Adult
14	Yes / No	□	□	□	□	□ < 20 seconds □ ≥ 20 seconds	□ M □ F	□ Child □ Juvenile □ Adult
15	Yes / No	□	□	□	□	□ < 20 seconds □ ≥ 20 seconds	□ M □ F	□ Child □ Juvenile □ Adult
16	Yes / No	□	□	□	□	□ < 20 seconds □ ≥ 20 seconds	□ M □ F	□ Child □ Juvenile □ Adult
17	Yes / No	□	□	□	□	□ < 20 seconds □ ≥ 20 seconds	□ M □ F	□ Child □ Juvenile □ Adult
18	Yes / No	□	□	□	□	□ < 20 seconds □ ≥ 20 seconds	□ M □ F	□ Child □ Juvenile □ Adult
19	Yes / No	□	□	□	□	□ < 20 seconds □ ≥ 20 seconds	□ M □ F	□ Child □ Juvenile □ Adult
20	Yes / No	□	□	□	□	□ < 20 seconds □ ≥ 20 seconds	□ M □ F	□ Child □ Juvenile □ Adult
21	Yes / No	□	□	□	□	□ < 20 seconds □ ≥ 20 seconds	□ M □ F	□ Child □ Juvenile □ Adult
22	Yes / No	□	□	□	□	□ < 20 seconds □ ≥ 20 seconds	□ M □ F	□ Child □ Juvenile □ Adult
23	Yes / No	□	□	□	□	□ < 20 seconds □ ≥ 20 seconds	□ M □ F	□ Child □ Juvenile □ Adult
